# PI3K/AKT/mTOR signaling participates in insulin‐mediated regulation of pathological myopia‐related factors in retinal pigment epithelial cells

**DOI:** 10.1186/s12886-021-01946-y

**Published:** 2021-05-17

**Authors:** Yunqin Li, Junliang Jiang, Jin Yang, Libo Xiao, Qiyun Hua, Yue Zou

**Affiliations:** 1 Ophthalmology Department, 2nd People’s Hospital of Yunnan Province, The Affiliated Hospital of Yunnan University, No. 176 Qingnian Road, Wuhua District, Yunnan Province 650021 Kunming, China; 2 Orthopedics and Traumatology Department, 2nd People’s Hospital of Yunnan Province, The Affiliated Hospital of Yunnan University, 650021 Kunming, Yunnan Province China

**Keywords:** Pathological myopia, Insulin, PI3K/AKT**/**mTOR, RPE cells

## Abstract

**Background:**

Insulin positively correlates with the length of the eye axis and is increased in the vitreous and serum of patients with pathological myopia (PM). How insulin influences the physiological process of retinal pigment epithelial (RPE) cells in PM remains unclear. This study aimed to explore the effect of insulin on the ultrastructure and function of RPE cells and the role of PI3K/AKT**/**mTOR signaling involved in the development of PM.

**Methods:**

The ARPE-19 cells were treated with different concentrations of insulin to analyze the cell morphology, cell viability, the protein level of insulin receptor β, and the mRNA and protein levels of and PM-related factors (TIMP-2, MMP-2, bFGF, and IGF-1). The ultrastructure of APRE-19 cells was also observed after insulin treatment. Besides, the PI3K/AKT/mTOR signaling was studied with or without the PI3K inhibitor LY294002 in ARPE-19 cells.

**Results:**

Insulin enhanced the cell viability of ARPE-19 cells and caused the endoplasmic reticulum to expand and vesiculate, suggesting increased secretion of growth factors and degeneration in ARPE-19 cells. Furthermore, the insulin receptor β was stimulated with insulin treatment, subsequently, the phosphorylation of AKT and mTOR was positively activated, which was adversely suppressed in the presence of LY294002. The secretion of TIMP-2 and bFGF was significantly decreased, and the secretion of MMP-2 and IGF-1 was highly elevated with insulin treatment depending on the concentration in ARPE-19 cells. Furthermore, the effect of insulin on PM-related proteins was restored with the addition of LY294002.

**Conclusions:**

Our results indicated that insulin regulated the secretion of PM-related factors via the PI3K/AKT**/**mTOR signaling pathway in retinal pigment epithelial cells, and thus probably promoted the development of PM through transducing regulation signals from retina to choroid and sclera.

## Background

Pathological myopia (PM) is defined as an eye axial length larger than 26 mm or a refractive error greater than 6 D, accompanied by posterior scleral staphyloma and chorioretinal degeneration in the macular area [1]. The development of this disease leads to irreversible damage to the retinal tissue and it is one of the leading causes of blindness over the world. The incidence of PM is 2 %, and it is increasing year by year [2]. A recent study found that systemic metabolic factors may be related to the abnormal growth of PM eyeballs [3]. Further research found that serum and vitreous insulin levels in PM patients increased [4].

RPE cells are one of the important cells in the initiation mechanism of the retina-choroid-sclera pathway [5]. Progression of PM has discovered that RPE cells may play an important role in the formation and development of myopia [6, 7]. For example, it has been shown that transforming growth factor and bone morphogenetic protein pathways are involved in the regulation of eye growth and myopia in RPE cells [6]. Previous studies have revealed that the growth of the eye axis and the deformation of the eyeball during PM are caused by the steady-state changes of eyeball growth, which are controlled by optical signals [8]. After receiving the optical signal, the local retina can control the growth of the local eyeball and the refractive state of the eyeball. The current research generally believes that the growth of the eyeball originates from the retina [9]. After the retina receives optical signals, it can release a variety of signal molecules to control the growth rate of the underlying tissues and affect the process of myopia [10]. In this signal transmission system, RPE cells are one of the most important cells and are closely related to the occurrence of eyeball growth and myopia. It is located in the outermost layer of the retina and separates the retina from the choroid and sclera. This key position of RPE between the retina and the choroid makes it a possible conduit for the growth regulation signal from the retina to the choroid and sclera. RPE has the function of secreting various cytokines and growth factors and carrying out ion transport [11]. In turn, RPE cells have a similar barrier function and prevent the active substances of the retina from reaching downstream tissues, thereby preventing changes in downstream tissue morphology and function, so the disordered secrete function in RPE cells is an important cause of changes in the eye axis. Therefore, it is believed that the active substance of the retina acts as a primary signal on RPE cells first, which will cause function changes in RPE cell, and then generate secondary signals downstream [10, 12].

Insulin is one of the indispensable hormones to promote tissue growth. It is a protein hormone secreted by insulin β cells and stimulated by endogenous or exogenous substances such as glucose, lactose, glucagon, etc. [13]. It is the only protein hormone in the body that lowers blood sugar and plays its role in promoting growth and inhibiting protein decomposition. In recent years, insulin has been used in animal models to study the mechanism of eyeball growth regulation [14]. Animal studies have found that the role of exogenous insulin in the formation of animal ocular myopia is mainly manifested in the injection of insulin into the eye, which can promote the growth of the eye axis and the thinning of choroidal tissue [15]. Further studies have proposed that a certain concentration of exogenous insulin can also act on the eyes of form-deprivation animal models, causing a further deepening of myopia and an increase in the length of the eye axis [4]. At present, the specific mechanism of insulin has not yet been clarified, and the study is limited to animal experiments, and no clinical studies have been reported.

Insulin receptors (INSR) are widely distributed in human eye tissues, including the retina, choroid, and sclera [16]. The signal transduction of insulin receptors mainly goes through two pathways, the PI3K pathway and the MAPK pathway [17]. Among them, the PI3K/AKT/mTOR pathway plays an important role in cellular sugar uptake, glycogen synthesis, protein synthesis, and cell survival [18]. Insulin binds to INSR, phosphorylates INSR substrate protein, triggers PI3K to activate AKT, activates mTOR through the TSC pathway, initiates cell growth regulation, and releases downstream MMP-2/TGF-β2 and other cytokines, which are involved in the regulation of eye growth [19]. However, whether insulin can regulate the biological behavior of RPE cells through this pathway in the eye, thereby regulating the growth of the eyeball, has not yet been reported.

Herein, we will investigate the mechanism of insulin in the occurrence and development of PM in RPE cells.

## Methods

### Cell culture

The ARPE-19 cells (ATCC, USA) were cultured in DMEM with 10 % fetal bovine serum (Gibco, USA) in a 37 °C incubator with 5 % CO_2_. The cells were cultured in serum-free DMEM for 24 h before treated with insulin. 0.1 µg/mL and 1 µg/mL concentrations of insulin were used to treat the ARPE-19 cells for 24 or 72 h in the study. 0 µg/mL of insulin stands for the blank control.

### CCK-8 assay

A CCK-8 kit (Cell Counting Kit-8, Beyotime, China) was used to detect ARPE-19 cell viability (with or without insulin treatment at the concentration of 0.1 or 1 µg/mL). Cells in the logarithmic growth phase were seeded into 96-well plates at a density of 5 × 10^4^/well and cultured overnight. At the time point of 72 h, 10 µL CCK-8 reagent was added to each well, and cells incubated for another 1 h. The absorbance of cells at 450 nm was measured using a microplate reader.

### Transmission electron microscopy

ARPE-19 cell suspensions at the concentration of 1×10^6^ were centrifuged at 200×g for 10 min, and then fixed with 3 % glutaraldehyde for 2 h at 4 °C and postfixed with 1 % osmium tetroxide (OsO_4_) for another 2 h at 4 °C. After that, the cells were dehydrated with ethyl alcohol series for 15 min and embedded in Epon. Ultrathin sections (60–70 nm) were stained with uranyl acetate for 8 min and lead citrate for 5 min. The ultrastructure of ARPE-19 cells was viewed using a transmission electron microscope (HD-2700, Hitachi, Japan). Images were captured from three different fields at a magnification of 6800×.

### Western blotting

The APRE-19 cells were washed 3 times with PBS and then lysed with radio immune precipitation (RIPA) buffer. The total protein was extracted in RIPA buffer, separated on polyacrylamide gels, and then immobilized on polyvinylidene fluoride (PVDF) membrane. Following blocking with 5 % non-fat milk at room temperature for 1.5 h, the PVDF membranes were incubated with primary antibodies against insulin receptor β, p-AKT, AKT, p-mTOR, mTOR, and β-actin for 12 h at 4 °C environments. The primary antibodies were purchased from Abcam and used at 1:1000 dilution. Then the PVDF membrane was washed 3 times with PBST. Subsequently, the membrane was incubated with a horseradish peroxidase-conjugated secondary antibody at a dilution of 1:5000 in a shaker at room temperature for 1 h. Finally, the ECL kit was used to process the membrane for the color reaction. The quantitative western blot results were normalized to the results of β-actin.

### RT-qPCR assay

Total RNA was extracted using Trizol reagent (Invitrogen, UK). The purity and concertation of extracted RNA were determined by the NanoDrop TM ND-1000 (Thermo Fisher Scientific, USA). Prime Script TM RT reagent Kit was used to prepare cDNA (Takara, Japan). RT-qPCR was performed using the SYBR Green PCR master mix (Takara, Japan). The RT-qPCR amplification was performed in triplicate, the expression of RNA was calculated using the 2-ΔΔCt method [20]. GAPDH expression was used as the internal control, allowing comparison of mRNA levels. Primers used in our study are listed in Table 1.

**Table 1:** List of primers used in reverse transcription-quantitative PCR

Gene name	Primer sequences (5’-3’)
TIMP-2-F	ACTGCAGGA TGGACTCTTGCA
TIMP-2-R	TTTCAGAGCCTTGGAGGAGCT
MMP-2–F	CCACTGCCTTCGATACAC
MMP-2-R	GAGCCACTCTCTGGAATCTTAAA
b-FGF-F	ACCCCGACGGCCGA
b-FGF-R	TCTTCTGCTTGAAGTTGTAGCTTGA
IGF-1-F	TGTGTGGAGACAGGGGCTTT
IGF-1-R	TTGGCAGGCTTGAGGGGT
GAPDH-F	ACGGCAAGTTCAACGGCACAG
GAPDH-R	GAAGACGCCAGTAGACTCCACGAC

### ELISA

Cells treated with or without insulin or LY294002 were seeded into 6-well plates. After incubation for 48 h, the protein levels of TIMP-1, MMP2, bFGF, and IGF-1 were detected in cell supernatants using an ELISA kit (Thermo Fisher Scientific, USA), according to the manufacturer’s protocol. In brief, the diluted samples were added to the monoclonal antibody-coated well plate. After incubation for 2 h at 37 °C, the plate was washed, and an enzyme-labeled antibody was added to each well except the blank wells. After further incubation for 1 h at 37 °C, the plate was washed 5 times. After patting dry, each well was added with color developer A and B. Color reaction was stopped after 15 min with the stop solution. The absorbance was measured at 450 nm with a multimode microplate reader (Thermo, MK-3).

### Statistical analysis

The data analysis was performed mainly using Graphpad Prism version 8.0 statistical software. All experiments were conducted at least three times. All data were presented as means ± standard deviations (SD). Student’s *t*-*test* or one-way analysis of variance (ANOVA) was performed to calculate the statistical differences. *P* < 0.05 was considered to indicate significance. Image J was used to carry out the semiquantitative analysis after the western blotting experiments.

## Results

### Insulin enhances the cell viability of ARPE-19 cells

**Fig. 1:**
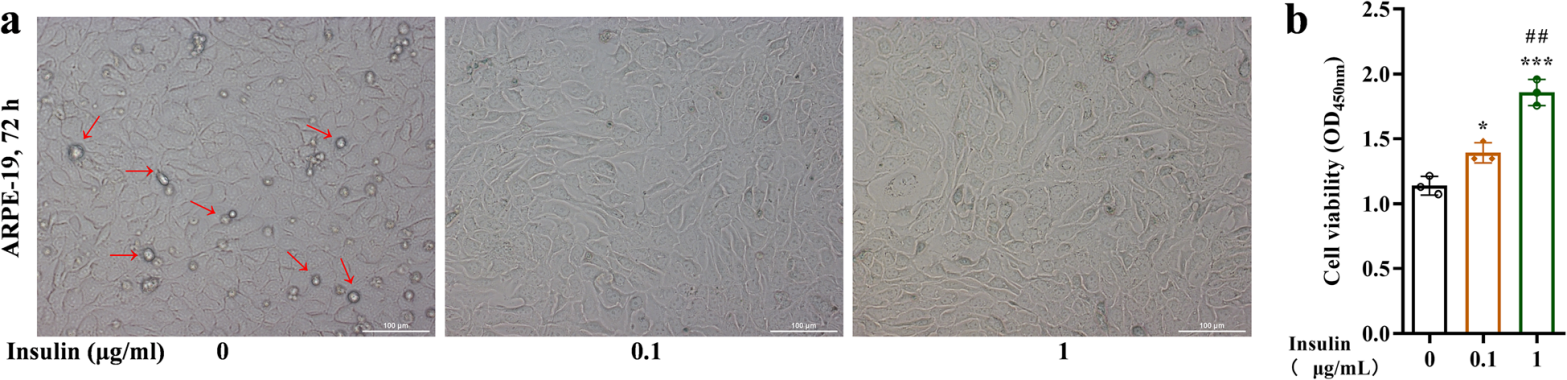
Insulin enhances the cell viability of ARPE-19 cells. Cell morphology (**a**) and cell viability (**b**) of ARPE-19 cells treated with insulin at different concentrations (0, 0.1, 1 µg/mL) for 72 h. The red arrows point to floating dead cells. *p < 0.05, ***p < 0.001 vs.. the insulin-free group; ##*p* < 0.01 vs.. the 1 µg/mL of insulin treatment group. Scale bar: 100 μm

APRE-19 cells were treated with different concentrations of insulin (0, 0.1, 1 µg/mL) for 72 h. The results in Fig. 1 a showed that the floating dead cells were significantly reduced after 72 h of insulin treatment. The number of dead cells decreased as the insulin concentration increased. The cell viability was also detected with CCK-8. The cell viability was increased with the insulin concentration rose (Fig. 1b).

### Endoplasmic reticulum expansion and vesiculation of the ARPE-19 cells after insulin treatment

**Fig. 2:**
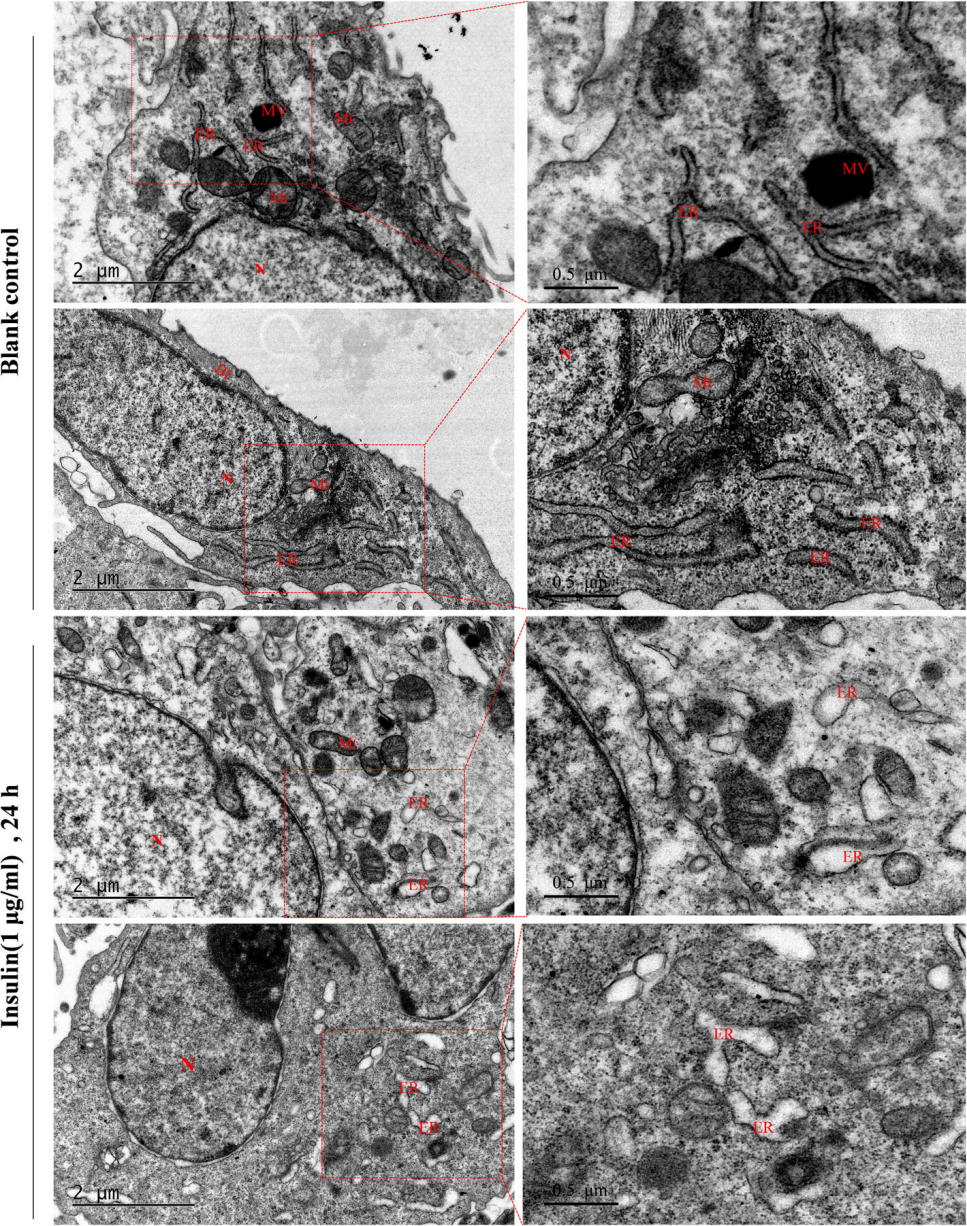
Ultrastructure of ARPE-19 cells after insulin treatment. The ARPE-19 cells were treated with 1 µg/mL of insulin for 24 h. Transmission electron microscopy of Nuclei (N), basal infoldings (BI), endoplasmic reticulum (ER), defective melanin vesicle (*), melanin vesicle (MV), and mitochondria (Mt) in ARPE-19 cells. Scale bars: 2 μm on the left pictures, 0.5 μm on the right pictures

The ultrastructure of ARPE-19 cells was observed in a transmission electron microscope after treatment with 1 µg/mL of insulin (Fig. 2). After 24 h of insulin treatment, the endoplasmic reticulum expanded and vesiculated to varying degrees, along with the particles on the membrane of the rough endoplasmic reticulum were mostly lost, suggesting increased secretion of cytokines and degeneration in ARPE-19 cells [21].

### Effects of insulin on insulin receptor β and factors involved in PM

**Fig. 3:**
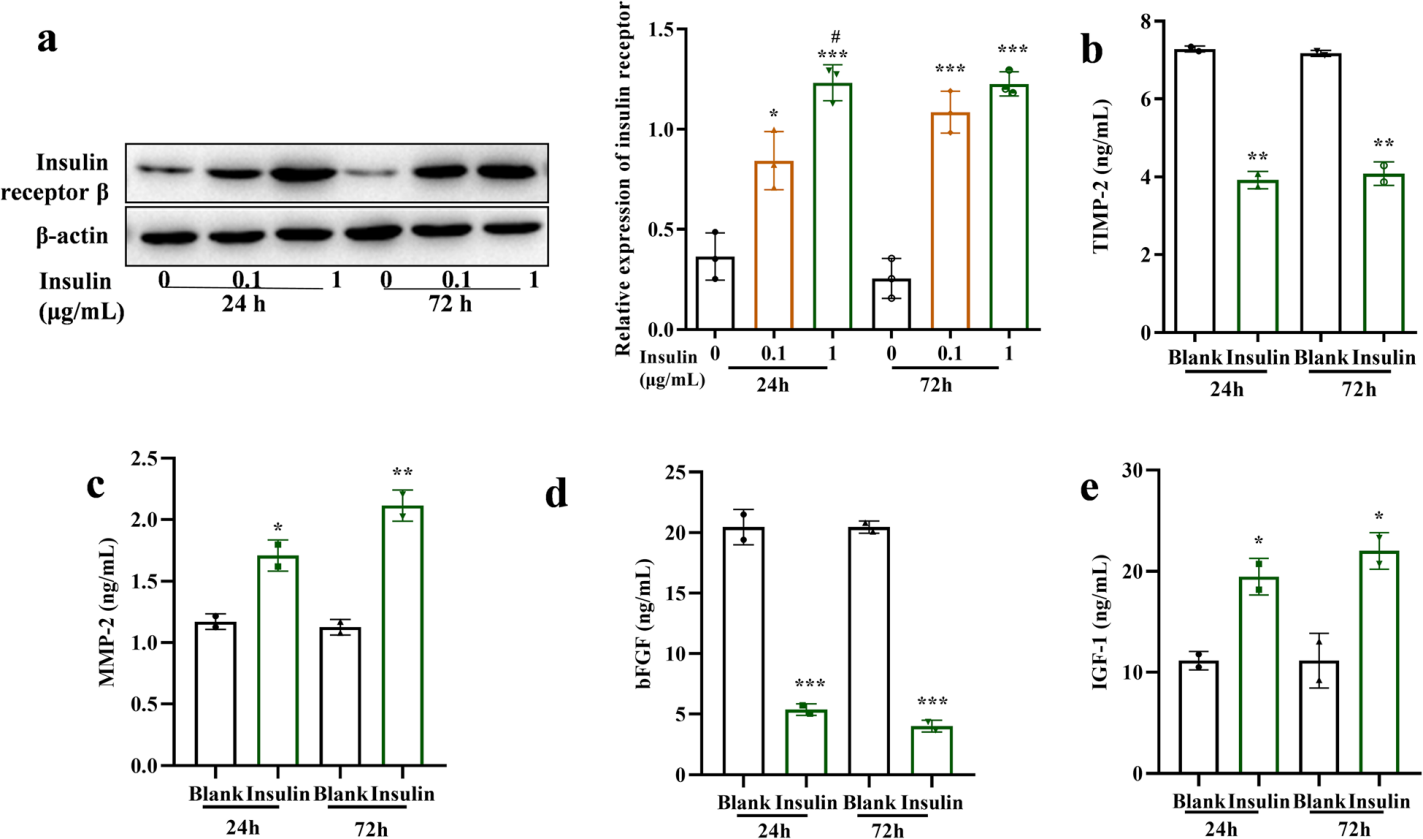
Insulin had effects on insulin receptor β and factors involved in PM. **a**. The protein expression level of insulin receptor β was measured by western blotting at different concentrations of insulin treatment, *p < 0.05, ***p < 0.001 vs.. the control group, #*p* < 0.05, vs.. the 0.1 µg/mL insulin group. The protein levels of (**b**) TIMP-1, (**c)** MMP2, (**d)** bFGF, and (**e)** IGF-1, involved in pathological myopia, were detected with ELISA assay in ARPE-19 cells treated with 1 µg/mL of insulin for 24 or 72 h. *p < 0.05, **p < 0.01, ***p < 0.001 vs.. the blank group

To further study the effect of insulin in PM, the protein expression level of insulin receptor β was measured by western blotting at different concentrations (0, 0.1, 1 µg/mL) of insulin treatment. The results showed that insulin dramatically promoted the INSRβ protein level (*p* < 0.05 at 0.1 µg/mL, and *p* < 0.001 at 1 µg/mL after 24 h insulin treatment; *p* < 0.001 both at 0.1 µg/mL and 1 µg/mL after 72 h insulin treatment). The INSRβ protein level was significantly higher with 1 µg/mL insulin treatment than that with 0.1 µg/mL insulin treatment after 24 h, whereas no significance was observed after 72 h (Fig. 3 a). The secretion of TIMP-1, MMP2, bFGF, and IGF-1, which were involved in pathological myopia, were detected with ELISA assay in ARPE-19 cells treated with 1 µg/mL of insulin for 24 or 72 h. The secretion of TIMP-2 and bFGF was significantly reduced in the insulin treatment group than in the blank control group (Fig. 3b and d). On the other hand, the secretion of MMP-2 and IGF-1 were increased in the insulin treatment group than in the blank control group (Fig. 3 c, e).

### The inhibitor of PI3K LY294002 acted an antagonistic effect on insulin

**Fig. 4:**
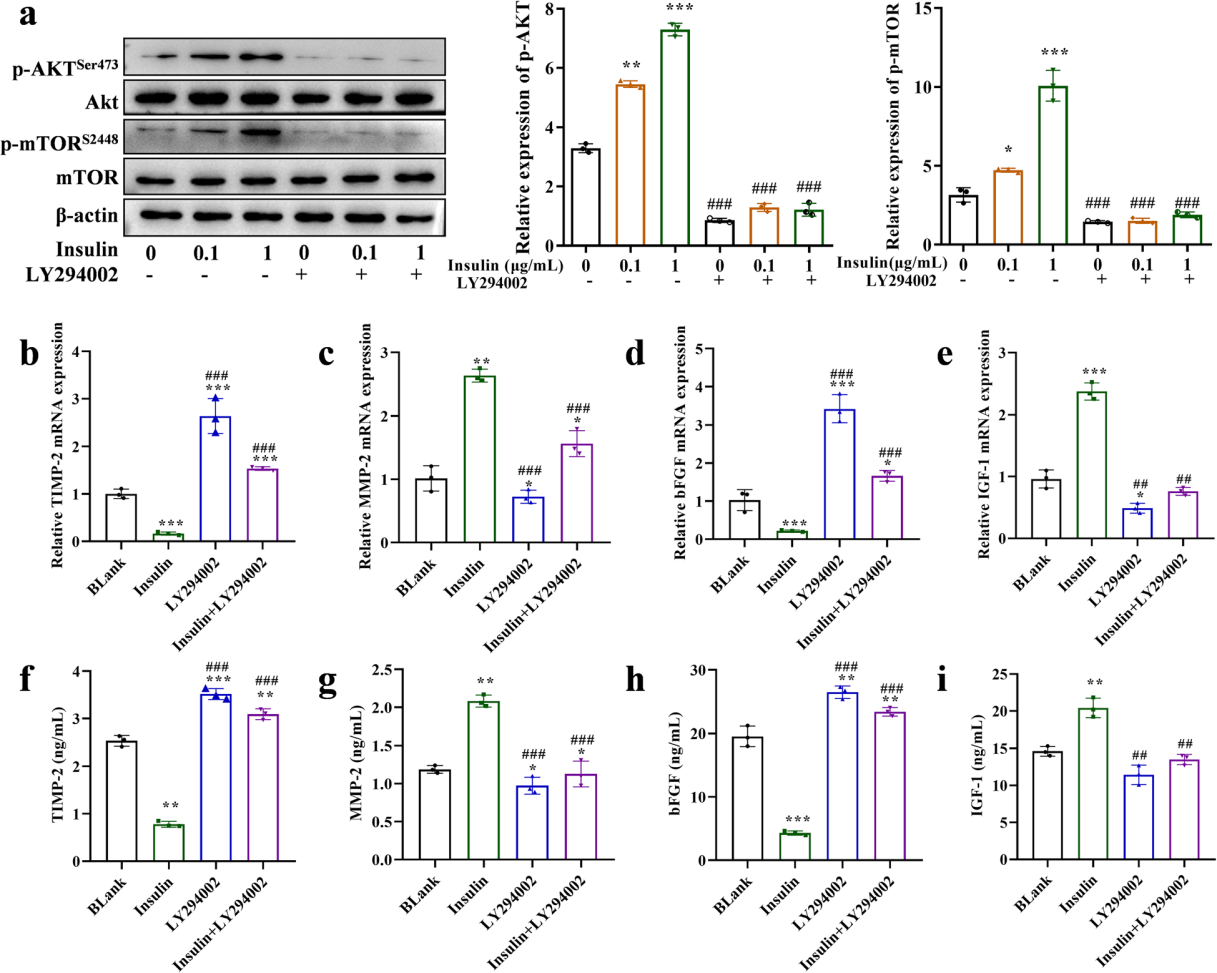
The inhibitor of PI3K LY294002 acted antagonistic effect on insulin. **a** The protein levels of main molecules in the PI3K/AKT/mTOR signaling pathway were measured by western blotting, *p < 0.05, **p < 0.01, ***p < 0.001 vs.. the control group, ### *p* < 0.0501, vs.. the 24 h treatment group. Transcriptional levels of (**b**) TIMP-2, (**c**) MMP-2, (**d**) bFGF, and (**e**) IGF-1 were detected by RT-qPCR in ARPE-19 cells treated with 1 µg/ml of insulin or LY294002 for 72 h. Protein expression levels of (**f**) TIMP-2, (**g**) MMP-2, (**h**) bFGF, and (**i**) IGF-1 were detected by ELISA in ARPE-19 cells treated with 1 µg/mL of insulin or LY294002 for 72 h. *p < 0.05, **p < 0.01, ***p < 0.001 vs.. the blank group; ##*p* < 0.01, ###*p* < 0.001 vs.. the Insulin group

To examine the role of PI3K/AKT/mTOR signaling in PM. The main proteins in the PI3K/AKT/mTOR signaling pathway were measured by western blotting with or without insulin treatment. The phosphorylation levels of AKT and mTOR were both markedly promoted with insulin treatment, especially at the concentration of 1 µg/mL (*p* < 0.001). However, this promotion with insulin treatment disappeared in the presence of LY294002 (Fig. 4 a). The mRNA and protein expression levels of TIMP-2, MMP-2, bFGF, and IGF-1 were also detected with LY294002 treatment for 72 h. The mRNA and protein expression levels of TIMP-2 and bFGF were significantly higher in the LY294002 treatment group than in the insulin treatment group (Fig. 4b, d, f and h). The mRNA and protein expression levels of MMP-2 and IGF-1 were lower in the LY294002 treatment group than in the insulin treatment group (Fig. 4c, e, g, and i). Our results showed that insulin activated INSR and then stimulated AKT phosphorylation, which subsequently activated mTOR phosphorylation. The PI3K inhibitor LRY294002 successfully restored the induction of the phosphorylation of AKT and mTOR.

## Discussion

RPE cells are the main source of many growth factors and cytokines, including insulin-like growth factor-1 (IGF-1), transforming growth factor-β (TGF-β), and basic fibroblast growth factor (bFGF). They are locally synthesized and subsequently secreted, and play important roles in maintaining the structure and homeostasis of the retina and choroid [6, 22]. Among the various cytokines secreted by RPE cells, TGF-β2 is a multifunctional cytokine that regulates cell growth and differentiation [23]. It is one of the key signal molecules that regulate the growth of the eyeball [24]. It can promote the proliferation of scleral cells and regulate the synthesis and degradation of the scleral extracellular matrix (ECM) [25]. Also, our previous study has proved that TGF-β2 is a myopic signal factor in RPE cells. Besides, we have validated that insulin can promote the proliferation of RPE cells and the secretion of TGF-β2 in RPE cells for signal transmission and promote the occurrence of myopia [26]. In our present study, the other two growth factors, IGF-1 and bFGF were studied. We found that insulin positively activated the proliferation of RPE cells, significantly promoted the secretion of IGF-1, and reduced the secretion of bFGF in ARPE-19 cells. As the insulin concentration increased and the action time was prolonged, the effect became more obvious. Our results proved that the myopia-promoting effect of insulin was likely to be through affecting RPE cells, promoting the increase of the secondary myopia signal molecule (TGF-β2, IGF-1, bFGF) secreted by RPE cells, and then acting on choroidal sclera and other downstream tissues, causing eye axis growth, and eventually promoting the occurrence of myopia.

The expression of MMP-2 (matrix metalloproteinases 2) and TIMP-2 (tissue inhibitors of metalloproteinase 2) were also detected in this study. MMP-2, which is a kind of zinc-dependent protease, can degrade the extracellular matrix of the sclera and mediate scleral remodeling in experimental myopia [27]. TIMPs are a group of endogenous inhibitors of MMPs, regulating the proteolytic activity of MMPs [28]. Pieces of evidence from myopic animal models (e.g. chicken and guinea pig) have shown an elevation of MMP-2 protein and mRNA levels in the sclera of myopic eyes, and a reduction of TIMP-2 expression [29, 30]. In our study, insulin significantly promoted MMP-2 mRNA and protein expression levels and decreased the mRNA and protein levels of TIMP-2 in ARPE-19 cells. As the insulin concentration increased and the action time was prolonged, the effect became more obvious. Our results were assistant with previous studies.

At the same time, we detected a high expression of the insulin receptor (INSR) after insulin stimulation in ARPE-19 cells. The insulin activates a complex intracellular signaling network through INSR and the classic PI3K and ERK cascade. However, in many cases, MAPK does not seem to be necessary for the insulin-mediated signaling pathway [31, 32]. Based on the results of previous experiments that the MAPK inhibitor PD98059 failed to cause significant changes with insulin treatment, the PI3K pathway was chosen in this study [33]. We found that the insulin receptor activated the phosphorylation of AKT and mTOR after insulin stimulation, and this effect was restored by the PI3K inhibitor LY294002. Besides, the expression levels of myopia-related factors were also restored by LY294002. The results showed that after stimulating the insulin receptor, insulin acted on RPE cells through the PI3K/AKT/mTOR signaling pathway.

We also observed the ultrastructure changes in the ARPE-19 cells after insulin treatment. In the process of cell degeneration and necrosis, the granular endoplasmic reticulum generally expands [34]. The lighter and limited expansion can only be seen under the electron microscope, and the severe expansion can be manifested under the optical microscope as vacuole formation. Besides, it has been reported that the endoplasmic reticulum vesiculation and expansion can lead to ER stress-induced apoptosis [21]. After 24 h of insulin treatment, expansion and vesiculation in the endoplasmic reticulum were observed in our experiment, suggesting increased secretion of cytokines and degeneration in ARPE-19 cells.

## Conclusions

In summary, our study suggested that insulin acted on RPE cells as a primary signal molecule, and then increased the cell viability and promoted the secretion of pathological myopia-related factors, which was accompanied by the ultrastructure alteration, through PI3K/AKT/mTOR signaling pathway. Our findings may provide some evidence for the pathogenesis of pathologic myopia and a target for its clinical treatment.

## Data Availability

All data generated or analyzed during this study are included in this article.

## Abbreviations

RPE: Retinal pigment epithelial
PM: Pathological myopia
INSR: Insulin receptor
ER: Endoplasmic reticulum
IGF-1: Insulin-like growth factor-1
TIMP: Tissue inhibitor of metalloproteinase
MMP: Matrix metalloproteinase
TGF-β: Transforming growth factor-β
bFGF: Basic fibroblast growth factor
